# Body composition paradox: high muscle mass and adiposity jointly predict incident chronic kidney disease in a Korean cohort

**DOI:** 10.1016/j.clinsp.2026.100879

**Published:** 2026-03-12

**Authors:** Eun Young Lee, Choon Hee Chung, Tae Hwa Go, Sang Baek Koh, Jung Ran Choi

**Affiliations:** a Division of Nephrology, Department of Internal Medicine, Soonchunhyang University Cheonan Hospital and Soonchunhyang University College of Medicine, Cheonan, Republic of Korea; b Department of Internal Medicine, Yonsei University Wonju College of Medicine, Wonju, Republic of Korea; c Center of Biomedical Data Science, Yonsei University Wonju College of Medicine, Wonju, Republic of Korea; d Department of Preventive Medicine, Yonsei University Wonju College of Medicine, Wonju, Republic of Korea; e Institute of Genomic Cohort, Yonsei University Wonju College of Medicine, Department of Biomedical Systems Informatics, Yonsei University College of Medicine, Republic of Korea

**Keywords:** Chronic kidney disease, Sarcopenic obesity, Adipokines, Bioelectrical impedance, Analysis, Cohort study, Korean genome and epidemiology study (KoGES)

## Abstract

**Objective:**

Diabetes, hypertension, and obesity are common traditional risk factors for Chronic Kidney Disease (CKD). In this population-based longitudinal study, the authors identified risk factors associated with CKD development.

**Method:**

A total of 6,238 Korean adults aged 40- to 69-years without baseline CKD were included. Among them, 4,057 participants with baseline estimated Glomerular Filtration Rate (eGFR) measurements were analyzed. Logistic regression models were used to evaluate significant predictors of CKD.

**Results:**

Over a 10-year follow-up period, CKD developed in 354 subjects (8.7 %). Leptin levels were significantly higher in participants who developed CKD compared to those who did not (8.89 ± 8.00 vs. 6.44 ± 5.52, *p* < 0.001). After adjusting for confounding factors, logistic regression analysis indicated that participants in the highest quartile of muscle mass were 2.314-times more likely to develop CKD compared to those in the lowest quartile (Odds Ratio [OR = 2.314]; 95 % Confidence Interval [95 % CI: 1.372–3.902]; p = 0.014). In multivariable-adjusted models, the OR for incident CKD when comparing the lowest to the highest quartiles of body fat was 2.166 in men; however, the significance disappeared after adjusting for eGFR. Among women, higher leptin levels and lower adiponectin levels were independently associated with incident CKD (*p* = 0.003 and *p* = 0.038, respectively), regardless of traditional CKD risk factors. Furthermore, compared to subjects without CKD, those with metabolic syndrome had an OR (95 % CI) of 5.256 (2.813–9.820) for incident CKD after controlling for confounding factors.

**Conclusion:**

These findings suggest that muscle mass and body fat may serve as better predictors of incident CKD.

## Highlights

• First large cohort study to identify high muscle mass as a novel and independent risk factor for incident CKD.
• Reveals a body composition paradox: both high adiposity and high muscle mass increase CKD risk.
• Gender-specific adipokine roles: Leptin (women) is a stronger predictor than adiponectin.
• Metabolic syndrome confers a 5-fold higher risk of developing CKD over 10-years.
• Findings challenge the traditional focus solely on fat and highlight muscle mass as a key prognostic factor.

## Introduction

Chronic Kidney Disease (CKD) is a clinical syndrome resulting from changes in kidney function and/or structure, characterized by its irreversibility and slow, progressive evolution. A key aspect of this pathology is its strong association with an increased risk of complications and mortality, particularly from cardiovascular causes^1^^,^^2^ Diabetes, hypertension, and obesity are major contributors to the global disease burden and the most common traditional risk factors for CKD^3^

According to data from the Third National Health and Nutrition Examination Survey (NHANES III), approximately 8.3 million (4.6 %) adults in the United States have CKD,^2^ In Korea, the estimated prevalence of CKD is 7.9 %^4^ Given these figures, identifying individuals at risk of CKD, as well as those in the early stages of the disease, is crucial for timely prevention and intervention. Since obesity and insulin resistance are linked to CKD,^5^^,^^6^ increasing attention has been directed toward the role of adipokines ‒ hormones secreted by adipose tissue ‒ in kidney disease.

In vitro and animal studies suggest that adipokines, including leptin and adiponectin, may mediate pathological and functional changes in the renal parenchyma^7^^,^^8^ Elevated leptin and reduced adiponectin levels have been associated with obesity, dyslipidemia, insulin resistance, hypertension, and inflammation,^9^^,^^10^ all of which contribute to CKD pathogenesis. Studies consistently show that high leptin levels correlate with CKD in both the general population and among diabetic and obese non-diabetic individuals^10^

However, the rapidly increasing prevalence of CKD cannot be fully explained by known risk factors such as diabetes and hypertension, suggesting the involvement of additional contributing variables^11^ Identifying modifiable predictors may help mitigate the growing burden of CKD and its associated economic and social costs^12^ This population-based longitudinal study aimed to identify risk factors for CKD development.

## Materials and method

### Study population

This study utilized data from individuals in the community-based cohort known as the Wonju-Pyengchang Cohort 2^nd^ Wave, which is part of the Korean Genome Epidemiology Study (KoGES). The Wonju-Pyengchang Cohort is a longitudinal survey supported by the Korean government, specifically by the Korean National Research Institute of Health, the Korean Centers for Disease Control and Prevention, and the Ministry of Health and Welfare. The cohort aims to investigate the genetic and environmental factors contributing to chronic diseases in Koreans.

The study included all adults residing in the rural areas of Wonju and Pyengchang in South Korea, where demographic changes are minimal^13^^,^^14^ Informed written consent was obtained from all participants at each visit. The study protocol was approved by the Ethics Committee of the Korean Centers for Disease Control and the Institutional Review Boards of Yonsei University Wonju College of Medicine. The study was conducted in accordance with the ethical standards outlined in the Declaration of Helsinki. For this study, incident Chronic Kidney Disease (CKD) was defined as an estimated Glomerular Filtration Rate (eGFR) below 60 mL/min per 1.73 m^2^, and a rapid decline in eGFR was defined as a decrease of more than 3 mL/min per 1.73 m^2^ per year. This study involved human participants and human data, and it includes a statement on ethics approval and consent, as well as the name of the ethics committee that approved the study. The study was approved by the Institutional Review Board of Wonju Christian Hospital in accordance with the Declaration of Helsinki. All participants provided informed written consent.

### Anthropometric and laboratory measurements

Anthropometric indices, including Body Mass Index (BMI), smoking status, regular physical exercise, and other relevant factors, were assessed. Waist circumference was measured using a tape measure (SECA-200, SECA, Hamburg, Germany). Systolic Blood Pressure (SBP) and Diastolic Blood Pressure (DBP) were measured twice using a standardized mercury sphygmomanometer (Baumanometer, Copiague, New York).

A venous blood sample was drawn from participants after a fasting period of more than 12-hours or overnight. Fasting glucose levels were measured using a glucose oxidase-based assay. Serum concentrations of Low-Density Lipoprotein (LDL) cholesterol, High-Density Lipoprotein (HDL) cholesterol, and Triglycerides (TGs) were analyzed using the enzymatic colorimetric method (Advia 1650, Siemens, Tarrytown, New York). Serum creatinine levels were determined using Jaffe’s method with a Hitachi Automatic Analyzer 7600 (Hitachi, Tokyo, Japan).

Serum aliquots were stored at -80°C until thawed for leptin analysis, which was conducted within one week after blood collection. Serum leptin concentrations were measured using Radioimmunoassay (RIA) (Linco Research, Inc.), with intra-assay and inter-assay Coefficients of Variation (CVs) ranging from 3.0 % to 6.2 %. Fasting insulin levels were determined using a double-antibody RIA assay (Biosource)^15^

Specifically, muscle mass was assessed using a bioelectrical impedance analysis device (InBody; InBody Japan Inc., Tokyo, Japan), which estimates body composition based on segmental multi-frequency analysis.

Alcohol consumption and smoking habits were assessed through self-administered questionnaires. Physical activity was categorized as either none/irregular (≤ 2 episodes per week) or regular (≥ 3 episodes per week). One episode of exercise was defined as engaging in physical activity for at least 30-minutes.

### Definition of metabolic syndrome (MetS)

According to the modified National Cholesterol Education Program Adult Treatment Panel III (NCEP-ATP III) criteria,^16^ Metabolic Syndrome (MetS) was defined as the presence of three or more of the following components: 1) Abdominal obesity, defined as a waist circumference ≥ 90 cm for males or ≥ 85 cm for females (following Korean specific cutoffs for abdominal obesity defined by the Korean Society of Obesity)^17^ 2) Hypertriglyceridemia, defined as a serum triglyceride concentration ≥ 150 mg/dL; 3) Low HDL cholesterol, defined as a serum HDL cholesterol concentration < 40 mg/dL for males or < 50 mg/dL for females; 4) High blood pressure, defined as a Systolic Blood Pressure (SBP) ≥ 130 mmHg, a Diastolic Blood Pressure (DBP) ≥ 85 mmHg, or treatment with antihypertensive agents; and 5) High fasting glucose, defined as a fasting serum glucose ≥ 100 mg/dL or previously diagnosed type 2 diabetes.

### Definition of incident CKD of estimated glomerular filtration rate (eGFR)

Estimated Glomerular Filtration Rate (eGFR) was calculated using the CKD-Epidemiology Collaboration (CKD-EPI) equation,^18^ eGFR (mL/min per 1.73 m^2^) = 141 × min (Scr/κ, 1)^α^ × max (Scr/κ,1)^- 1.209^ × 0.993^age (years)^ × 1.018 (if female) × 1.159 (if black), where κ is 0.7 for females and 0.9 for males, α is -0.329 for females and -0.411 for males, min indicates the minimum of Scr/κ or 1, and max indicates the maximum of Scr/κ or 1. Incident CKD was defined as Egfr < 60 mL/min per 1.73 m^2^ with a baseline eGFR of ≥ 60 ml/min per 1.73 m^2^ at the 6th consecutive follow-up^19^ The average annual rate of change in eGFR observed during follow-up was determined by dividing the difference in eGFR by the total years of follow-up between baseline and 10-years later.

### Statistical analyses

Data were analyzed and presented according to the characteristics of the variables. Continuous variables were expressed as mean ± standard deviation, while categorical variables were presented as frequency and percentage. Appropriate statistical tests were used to compare two groups, including the two-sample *t*-test for continuous variables and the Chi-Square test for categorical variables. Specifically, leptin, adiponectin, muscle mass, and body fat levels were each divided into four groups based on the 25^th^, 50^th^, and 75^th^ percentiles of the study population distribution. Accordingly, participants were categorized as follows: Q1 (≤ 25^th^ percentile), Q2 (25^th^–50^th^ percentile), Q3 (50^th^–75^th^ percentile), and Q4 (> 75^th^ percentile).

Logistic regression analysis was performed to identify factors associated with incident CKD, and results were reported as Odds Ratios (ORs) with 95 % Confidence Intervals (95 % CIs). A p-value < 0.05 was considered statistically significant. All statistical analyses were conducted using SPSS software, version 23.0 (SPSS Inc., Chicago, IL, USA).

The statistical models used for analysis were as follows: Model 1: Adjusted for age and gender; Model 2: Further adjusted for smoking status, alcohol consumption, and regular exercise; Model 3: Additionally adjusted for C-reactive protein, baseline BMI, baseline eGFR, diabetes, hypertension, and total cholesterol.

## Results

### Participant characteristics

Table 1 presents the baseline clinical and biochemical characteristics of the participants, categorized based on incident CKD status. Among the 4,057 subjects, 354 individuals (8.7 %) developed CKD during the 10-year follow-up period.

**Table 1:** Basic characteristics according to incident CKD.

Baseline variables	Incidence CKD(-)	Incidence CKD(+)	p-value
	n = 3703 (91.3 %)	n = 354 (8.7 %)	
Age (years)	54.62 ± 8.07	61.45 ± 6.78	<0.001
Gender (male, %)	1548 (41.8 %)	133 (37.6 %)	0.122
BMI (kg/m^2^)	24.60 ± 3.10	25.17 ± 3.27	0.268
Waist circumference (cm)	83.47 ± 8.76	85.72 ± 8.64	0.716
Systolic BP (mmHg)	130.12 ± 18.30	137.86 ± 18.92	0.066
Diastolic BP (mmHg	82.76 ± 11.71	84.75 ± 11.48	0.605
Fasting glucose (mg/dL)	95.76 ± 18.84	104.24 ± 33.46	<0.001
Total cholesterol (mg/dL)	200.27 ± 37.75	207.78 ± 41.88	0.007
HDL cholesterol (mg/dL)	46.07 ± 10.96	43.70 ± 9.93	0.213
LDL cholesterol (mg/dL)	117.04 ± 32.18	122.12 ± 34.97	0.124
Triglyceride (mg/dL)	147.06 ± 109.00	167.63 ± 102.18	0.920
HbA1C ( %)	5.58 ± 0.73	6.02 ± 1.15	<0.001
Creatinine (mg/dL)	0.93 ± 0.14	1.11 ± 0.56	<0.001
Baseline eGFR (mL/min/1.73 m^2^)	79.64 ± 10.58	63.71 ± 12.28	0.268
Uric acid (mg/dL)	4.86 ± 1.33	5.47 ± 1.72	<0.001
hsCRP (mg/dL)	1.93 ± 4.93	2.56 ± 4.69	0.041
Current drinker ( %)	1587 (43.0 %)	119 (33.6 %)	0.008
Current smoker ( %)	625 (16.9 %)	35 (9.9 %)	0.003
Hypertension ( %)	1968 (53.1 %)	245 (69.2 %)	<0.001
Diabetes ( %)	637 (17.2 %)	107 (30.3 %)	<0.001
Dyslipidemia ( %)	2276 (61.5 %)	263 (74.3 %)	<0.001
Regular exercise ( %)	1152 (31.2 %)	123 (34.8 %)	0.165
Adiponectin (ng/mL)	10008.92 ±5055.71	9997.39±5002.19	0.724
Leptin (ng/mL)	6.44±5.52	8.89±8.00	<0.001
Protein intake (g)	7.50±0.42	7.54±0.47	0.005
Muscle (kg)	40.87±7.28	40.04±7.20	0.613
Body Fat (kg)	18.20±5.11	19.08±4.92	0.271

CKD, Chronic Kidney Disease; BMI, Body Mass Index; BP, Blood Pressure; HDL, High-Density Lipoprotein; LDL, Low-Density Lipoprotein; hsCRP, High-Sensitivity C-Reactive Protein; eGFR, estimated Glomerular Filtration Rate.

Participants who developed CKD were older and had significantly higher levels of fasting glucose, total cholesterol, HbA1c, creatinine, and high-sensitivity C-Reactive Protein (hsCRP) compared to those who did not. Additionally, leptin levels were significantly higher in participants who developed CKD (8.89±8.00) than in those who did not (6.44 ± 5.52) (*p* < 0.001). Furthermore, baseline body fat was greater in participants who developed CKD (19.08 ± 4.92 kg) compared to those who remained CKD-free (Table 1).

### Risks of Incident CKD according to leptin, adiponectin, muscle and body fat

After adjusting for age, gender, smoking status, alcohol intake, and regular exercise, leptin was significantly associated with an increased risk of CKD, with an Odds Ratio (OR) of 3.94. Logistic regression analysis indicated that participants in the highest quartile of the muscle were 2.314 times more likely to develop CKD compared to those in the lowest quartile (OR = 2.314; 95 % CI: 1.372–3.902; *p* = 0.014) (Table 2). In both crude and adjusted conditional logistic regression models, higher baseline body fat was significantly associated with CKD in a dose-dependent manner. The OR for CKD in the highest versus lowest quartile of body fat was 1.661 (95 % CI: 1.163–2.372; *p* < 0.0001) (Table 2).

**Table 2:** Odds ratio of incident CKD according to leptin, adiponectin, muscle and body fat.

	Q1	Q2	Q3	Q4	p-value
**Leptin (ng/mL)**	30 (14.2 %)	59 (28.0 %)	45 (21.3 %)	77 (36.5 %)	<0.001
Crude OR	1	1.955 (1.243‒3.074)	1.473 (0.916‒2.367)	2.688 (1.738‒4.157)	<0.001
Model 1	1	2.461 (1.513‒4.003)	2.066 (1.155‒3.693)	3.848 (2.152‒6.800)	<0.001
Model 2	1	2.554 (1.558‒4.187)	2.106 (1.171‒3.789)	3.941 (2.190‒7.091)	<0.001
Model 3	1	1.602 (0.916‒2.802)	1.151 (0.573‒2.313)	1.461 (0.664‒3.216)	0.263
**Adiponectin**	76 (24.8 %)	81 (26.4 %)	75 (24.4 %)	75 (24.4 %)	0.950
Crude OR	1	1.083 (0.780‒1.502)	0.989 (0.709‒1.380)	1.022 (0.732‒1.426)	0.950
Model 1	1	0.912 (0.647‒1.286)	0.712 (0.498‒1.018)	0.596 (0.413‒0.860)	0.023
Model 2	1	0.894 (0.633‒1.263)	0.692 (0.483‒0.992)	0.582 (0.403‒0.841)	0.017
Model 3	1	0.903 (0.610‒1.334)	0.734 (0.486‒1.109)	0.795 (0.519‒1.217)	0.491
**Muscle (kg)**	104 (29.7 %)	82 (23.4 %)	86 (24.6 %)	78 (22.3 %)	0.098
Crude OR	1	0.754 (0.556‒1.021)	0.794 (0.588‒1.072)	0.694 (0.510‒0.944)	0.099
Model 1	1	1.107(0.804‒1.524)	1.729(1.157‒2.583)	2.400 (1.429‒4.033)	0.009
Model 2	1	1.096 (0.796‒1.509)	1.685 (1.126‒2.522)	2.314 (1.372‒3.902)	0.014
Model 3	1	0.861 (0.591‒1.255)	1.222 (0.722‒2.067)	0.955 (0.464‒1.964)	0.331
**Body fat (kg)**	63 (18.0 %)	85 (24.3 %)	104 (29.7 %)	98 (28.0 %)	0.010
Crude OR	1	1.321 (0.942‒1.852)	1.691 (1.221‒2.343)	1.557 (1.120‒2.165)	0.010
Model 1	1	1.552 (1.091‒2.207)	1.852 (1.314‒2.610)	1.683 (1.181‒2.397)	0.004
Model 2	1	1.515 (1.062‒2.162)	1.791 (1.267‒2.533)	1.661 (1.163‒2.372)	0.008
Model 3	1	1.121 (0.730‒1.723)	0.995 (0.603‒1.641)	0.864 (0.440‒1.694)	0.731

Results are described as odds ratio and 95 % Confidence Interval.

Model 1 was adjusted for age and gender.

Model 2: Model 1 + smoking status, alcohol intake and regular exercise.

Model 3: Model 2 + C-reactive protein, BMI, eGFR (baseline), DM, HTN, total cholesterol.

In multivariable-adjusted models comparing the lowest to the highest quartiles of body fat, the OR for incident CKD in men was 2.166 (95 % CI: 1.222–3.838; *p* = 0.030). However, this association lost significance after adjusting for baseline eGFR (Table 3). Among women, higher leptin levels and lower adiponectin levels were independently associated with incident CKD, even after adjusting for traditional CKD risk factors (*p* = 0.003 and *p* = 0.038, respectively) (Table 4). The odds of developing CKD in the highest quartile of leptin compared to the lowest quartile were 4.033 (95 % CI: 1.763–9.227; p for trend 0.005) in men and 2.124 (95 % CI: 1.293–3.490; p for trend 0.003) in women (Table 3, Table 4, respectively).

**Table 3:** Odds ratio of incident CKD according to leptin, adiponectin, muscle and body fat in men.

	Q1	Q2	Q3	Q4	p-value
**Leptin (ng/mL)**	8 (11.0 %)	16 (21.9 %)	22 (30.1 %)	27 (37.0 %)	0.015
Crude OR	1	1.906 (0.802‒4.528)	2.699 (1.181‒6.170)	3.362 (1.500‒7.537)	0.020
Model 1	1	2.093 (0.873‒5.017)	3.285 (1.420‒7.600)	4.001 (1.765‒9.073)	0.005
Model 2	1	1.960 (0.807‒4.759)	3.319 (1.420‒7.757)	4.033 (1.763‒9.227)	0.005
Model 3	1	2.141 (0.793‒5.783)	2.317 (0.842‒6.379)	2.653 (0.890‒7.910)	0.341
**Adiponectin**	29 (25.7 %)	26 (23.0 %)	30 (26.5 %)	28 (24.8 %)	0.947
Crude OR	1	0.902 (0.521‒1.562)	1.058 (0.622‒1.800)	1.026 (0.598‒1.761)	0.947
Model 1	1	0.763 (0.433‒1.344)	0.781(0.450‒1.355)	0.619 (0.351‒1.091)	0.428
Model 2	1	0.744(0.419‒1.322)	0.768 (0.440‒1.340)	0.605 (0.338‒1.082)	0.406
Model 3	1	0.766 (0.400‒1.466)	0.810 (0.422‒1.556)	0.788 (0.395‒1.570)	0.856
**Muscle (kg)**	38 (29.2 %)	25 (19.2 %)	33 (25.4 %)	34 (26.2 %)	0.272
Crude OR	1	0.592 (.350‒1.000)	0.814 90.499‒1.326)	0.793 (0.488‒1.286)	0.277
Model 1	1	0.820 (0.478‒1.407)	1.321 (0.793‒2.201)	1.694 (1.006‒2.852)	0.056
Model 2	1	0.753 (0.435‒1.303)	1.134 (0.672‒1.913)	1.544 (0.907‒2.626)	0.092
Model 3	1	0.607 (0.320‒1.152)	0.594 (0.305‒1.157)	0.771 (0.354‒1.678)	0.320
**Body fat (kg)**	21 (16.2 %)	29 (22.3 %)	38 (29.2 %)	42 (32.3 %)	0.091
Crude OR	1	1.293 (0.724‒2.308)	1.716 (0.988‒2.979)	1.872 (1.088‒3.222)	0.096
Model 1	1	1.583 (0.874‒2.865)	2.287 (1.295‒4.038)	2.529 (1.444‒4.430)	0.006
Model 2	1	1.313 (0.714‒2.414)	1.909 (1.069‒3.411)	2.166 (1.222‒3.838)	0.030
Model 3	1	0.895 (0.427‒1.876)	1.047 (0.457‒2.401)	0.929 (0.331‒2.606)	0.956

Results are described as odds ratio and 95 % Confidence Interval.

Model 1 was adjusted for age.

Model 2: Model 1 + smoking status, alcohol intake and regular exercise.

Model 3: Model 2 + C-reactive protein, BMI, eGFR (baseline), DM, HTN, total cholesterol.

**Table 4:** Odds ratio of incident CKD according to leptin, adiponectin, muscle and body fat in women.

	Q1	Q2	Q3	Q4	p-value
**Leptin (ng/mL)**	30 (21.7 %)	25 (18.1 %)	31 (22.5 %)	52 (37.7 %)	0.003
Crude OR	1	0.831 (0.480‒1.440)	0.999 (0.593‒1.682)	1.842 (1.148‒2.954)	0.004
Model 1	1	0.956 (0.542‒1.688)	1.150 (0.670‒1.974)	2.080 (1.269‒3.409)	0.004
Model 2	1	0.947 (0.535‒1.674)	1.133 (0.659‒1.948)	2.124 (1.293‒3.490)	0.003
Model 3	1	0.803 (0.424‒1.523)	0.777 (0.406‒1.488)	1.048 (0.506‒2.169)	0.667
**Adiponectin**	54 (27.8 %)	52 (26.8 %)	42 (21.6 %)	46 (23.7 %)	0.517
Crude OR	1	0.941 (0.630‒1.406)	0.740 (0.485‒1.129)	0.838 (0.554‒1.266)	0.519
Model 1	1	0.897 (0.589‒1.367)	0.631 (0.406‒0.981)	0.596 (0.386‒0.918)	0.048
Model 2	1	0.869 (0.569‒1.326)	0.613 (0.393‒0.954)	0.581 (0.376‒0.897)	0.038
Model 3	1	0.885 (0.550‒1.423)	0.654 (0.394‒1.085)	0.785 (0.475‒1.298)	0.405
**Muscle (kg)**	62 (28.2 %)	61 (27.7 %)	46 (20.9 %)	51 (23.2 %)	0.214
Crude OR	1	0.967 (0.665‒1.405)	0.700 (0.469‒1.044)	0.765 (0.518‒1.129)	0.271
Model 1	1	1.379 (0.932‒2.040)	1.171 (0.769‒1.783)	1.698 (1.111‒2.594)	0.084
Model 2	1	1.405 (0.948‒2.083)	1.188 (0.779‒1.811)	1.717 (1.123‒2.624)	0.074
Model 3	1	1.141 (0.722‒1.805)	0.933 (0.561‒1.550)	1.244 (0.687‒2.256)	0.671
**Body fat (kg)**	45 (20.5 %)	49 (22.3 %)	67 (30.5 %)	59 (26.8 %)	0.112
Crude OR	1	1.032 (0.677‒1.574)	1.520 (1.023‒2.261)	1.289 (0.859‒1.935)	0.115
Model 1	1	1.098 (0.709‒1.700)	1.468 (0.972‒2.217)	1.252 (0.822‒1.907)	0.272
Model 2	1	1.091 (0.705‒1.691)	1.478 (0.977‒2.235)	1.252 (0.821‒1.909)	0.255
Model 3	1	0.846 (0.495‒4.449)	0.875 (0.458‒1.671)	0.698 (0.280‒1.737)	0.844

Results are described as odds ratio and 95 % Confidence Interval.

Model 1 was adjusted for age.

Model 2: Model 1 + smoking status, alcohol intake and regular exercise.

Model 3: Model 2 + C-reactive protein, BMI, eGFR (baseline), DM, HTN, total cholesterol.

Compared to participants without CKD, those with metabolic syndrome (MetS) had a significantly higher risk of developing CKD (OR = 5.256; 95 % CI: 2.813–9.820) after controlling for confounding factors (Supplementary Tables). Additionally, the incidence of CKD progressively increased as the number of MetS components increased over the follow-up period for both men and women (p for trend = 0.001 and < 0.01, respectively).

## Discussion

In this prospective community-based cohort study of Korean adults, the authors observed a significant association between muscle mass, body fat, and the development of CKD, independent of conventional CKD risk factors. The risk of CKD increased progressively with the number of metabolic syndrome components. Additionally, the present study revealed that leptin was associated with an increased risk of CKD development over the 10-year follow-up period.

The risk factors for CKD are diverse and include developmental, physical, social, cultural, structural, environmental, and genetic factors^3^ In a multiethnic population study by Hsu et al.,^20^ BMI was strongly correlated with the risk of CKD. They found that individuals with a BMI > 40 kg/m^2^ had a seven-fold higher risk of CKD compared to those with a normal BMI^21^^,^^22^ Traditionally, leptin has been implicated in the progression of kidney disease through endothelial cell proliferation and mesangial cell hypertrophy. These results suggest a distinctive association between visceral and subcutaneous fat and inflammatory adipokines, such as leptin and adiponectin, in the general population^23^ In a cohort of men and women with varying degrees of CKD, higher volumes of Visceral Adipose Tissue (VAT) and Subcutaneous Adipose Tissue (SAT), as indicated by intrahepatic fat, were associated with a higher inflammatory burden, an altered adipokine profile, lower HDL cholesterol, and higher triglyceride levels. These associations were similar to those observed with other traditional measures of adiposity, such as BMI and Waist Circumference (WC), in relation to inflammation, insulin resistance, and adipokines^24^ Furthermore, kidney disease is associated with significant muscle impairment and functional limitations, contributing to a high prevalence of frailty. CKD patients often experience substantial muscle loss, weakness, and poor physical performance^25^ Therefore, the present study highlights the significant associations between body fat. Muscle mass and the risk of CKD.

CKD leads to the accumulation of uremic solutes, which disrupt skeletal muscle function and contribute to decreased physical performance and mobility limitations. The impairment of muscle mitochondrial energetics in CKD can result in detectable functional limitations. Specifically, the impaired coupling of ATP production to oxygen consumption within skeletal muscle mitochondria is associated with oxidative stress, metabolic dysfunction, and reduced exercise efficiency^26^ However, the mechanisms underlying muscle mass impairment and CKD development in humans remain poorly understood^27^ Skeletal muscle mass loss and dysfunction are common features not only in CKD but also in other chronic conditions such as cancer, diabetes mellitus, heart failure, and aging^28^, ^29^, ^30^ In healthy adults, whole-body protein undergoes continuous turnover, involving degradation into amino acids followed by the synthesis of new proteins. In a 60 kg man, the estimated daily protein turnover is approximately 250–300g, with skeletal muscle contributing around 100–120g of this amount^31^ Muscle mass is typically maintained through a tightly regulated balance between protein synthesis and degradation. However, even minor but sustained imbalances in these processes can lead to progressive muscle wasting over time. In CKD, muscle loss is predominantly driven by a disruption in the balance between anabolic and catabolic pathways that govern muscle homeostasis. The underlying molecular mechanisms include dysregulated protein metabolism, characterized by increased protein degradation and decreased protein synthesis, as well as impaired muscle regeneration^32^, ^33^ Several key cellular signaling pathways have been identified as regulators of muscle homeostasis. Among these, the Insulin-like Growth Factor-1 (IGF-1) pathway plays a pivotal anabolic role by enhancing protein synthesis and facilitating the recruitment and activation of muscle satellite cells. In contrast, the myostatin pathway acts as a catabolic regulator by promoting muscle protein degradation and suppressing satellite cell function^34^ The adverse effects of visceral fat are attributed to various mechanistic pathways^23^ Studies such as the Framingham Heart Study and the Dallas Heart Study have demonstrated that higher VAT and SAT volumes are associated with decreased total adiponectin levels and increased systemic inflammation markers, such as IL-6 and C-reactive protein^23^^,^^35^ VAT has also been linked to lower adiponectin levels, enhanced insulin resistance, and abnormal HDL cholesterol levels, whereas SAT has been associated with increased leptin and inflammatory markers but not with insulin resistance or dyslipidemia^36^ These findings enhance our understanding of the complex associations observed in individuals with CKD. Interestingly, these associations appear to be consistent across genders in this population, in contrast to patterns observed in the general population. It is important to note that the cohort was older and had a higher comorbidity burden compared to other study cohorts^23^

However, this study has some limitations. The results were based on a single measurement of leptin and adiponectin levels, which may be subject to random measurement errors and could have led to an underestimation of the strength of the correlations. Additionally, the generalizability of these findings may be limited to other populations, particularly those with different ethnic backgrounds, higher obesity levels, or a younger mean age. Furthermore, the authors were unable to find sufficient references to fully support the association between body fat, muscle mass, and the onset of CKD observed in this study.

## Conclusion

To the best of our knowledge, this study is the first and largest population-based prospective study to demonstrate that both muscle mass and body fat independently contribute to an increased risk of incident CKD. The study’s prospective design, the consistency of the observed associations, and the presence of graded dose-response associations provide strong evidence for the significant roles of muscle mass and body fat in CKD development in the general population. These findings underscore the importance of considering both muscle mass and body fat as key factors in predicting future CKD incidence.

## Data availability statement

All data generated or analyzed during this study are included in this article. Further enquiries can be directed to the corresponding author.

## Statement of ethics

This study included human participants and human data, a statement on ethics approval and consent, and the name of the ethics committee that approved the study. This study was approved by the Institutional Review Board of the Wonju Christian Hospital, according to the Helsinki Declaration. All the participants provided informed written consent.

This observational cohort study should follow the STROBE Statement.

## Author's contribution

Eun Young Lee: Original draft; writing-review, Funding acquisition & editing.

Choon Hee Chung: Writing-review & editing; supervision.

Tae Hwa Go: Conceptualization, Data curation, Visualization & Methodology.

Sang Baek Koh: Project administration; Validation; & editing; supervision.

Jung Ran Choi: Writing - original draft; Writing – review, conceptualization; supervision.

## Declaration of competing interest

The authors declare that they have no known competing financial interests or personal relationships that could have appeared to influence the work reported in this paper.

## Appendix Supplementary materials

Image, application 1
