# Supplementary material for: Body composition paradox: high muscle mass and adiposity jointly predict incident chronic kidney disease in a Korean cohort
Source: Clinics (Sao Paulo). 2026 Mar 12;81:100879. doi: 10.1016/j.clinsp.2026.100879 (PMC12997199; doi:10.1016/j.clinsp.2026.100879)
Supplement: Supplementary file 1 [file mmc1.docx]

CLINICS-D-25-00252_Supplementary Materials

**Supplementary Table 1** Association of CKD incident and the components of metabolic syndrome in baseline.

| **Nº of the components** | **Total** | **Men** | **Women** |
| --- | --- | --- | --- |
| 0 | 1.0 | 1.0 | 1.0 |
| 1 | 1.513 (0.870‒2.633) | 3.039 (0.887‒10.414) | 1.178 (0.627‒2.213) |
| 2 | 2.139 (1.256‒3.645) | 4.639 (1.405‒15.311) | 1.607 (0.874‒2.952) |
| 3 | 2.852 (1.678‒4.846) | 5.721 (1.739‒18.821) | 2.273 (1.240‒4.166) |
| 4 | 3.994 (2.314‒6.896) | 8.076 (2.412‒27.043) | 3.160 (1.686‒5.924) |
| 5 | 5.256 (2.813‒9.820) | 9.278 (2.541‒33.872) | 4.727 (2.237‒9.988) |
| p for trend | <0.001 | 0.001 | <0.001 |

**Supplementary Table 2** Odds ratio of incident CKD according to leptin, adiponectin, muscle and bodyfat in men.

|  | **Q1** | **Q2** | **Q3** | **p-value** |
| --- | --- | --- | --- | --- |
| **Leptin (ng/mL)** | 10 (13.7%) | 23 (31.5%) | 40 (54.8%) | <0.001 |
| Crude OR | 1 | 2.275 (1.068‒4.847) | 4.138 (2.037‒8.402) | <0.001 |
| Model 1 | 1 | 2.703 (1.255‒5.820) | 5.053 (2.457‒10.391) | <0.001 |
| Model 2 | 1 | 2.641 (1.209‒5.767) | 5.260 (2.527‒10.947) | <0.001 |
| Model 3 | 1 | 2.808 (1.147‒6.874) | 3.856 (1.520‒9.779) | 0.017 |
| **Adiponectin** | 36 (31.9%) | 40 (35.4%) | 37 (32.7%) | 0.920 |
| Crude OR | 1 | 1.102 (0.690‒1.759) | 1.059 (0.658‒1.706) | 0.921 |
| Model 1 | 1 | 1.000 (0.618‒1.618) | 0.727 (0.442‒1.194) | 0.339 |
| Model 2 | 1 | 0.979 (0.602‒1.595) | 0.728 (0.439‒1.206) | 0.381 |
| Model 3 | 1 | 1.028 (0.585‒1.808) | 0.932 (0.514‒1.689) | 0.942 |
| **Muscle (kg)** | 42 (32.3%) | 41 (31.5%) | 47 (36.2%) | 0.849 |
| Crude OR | 1 | 0.931 (0.595‒1.457) | 1.057 (0.685‒1.631) | 0.850 |
| Model 1 | 1 | 1.282 (0.807‒2.038) | 2.084 (1.306‒3.324) | 0.007 |
| Model 2 | 1 | 1.126 (0.701‒1.810) | 1.848 (1.147‒2.977) | 0.026 |
| Model 3 | 1 | 0.698 (0.395‒1.234) | 1.071 (0.550‒2.088) | 0.237 |
| **Body fat (kg)** | 30 (23.1%) | 42 (32.3%) | 58 (44.6%) | 0.019 |
| Crude OR | 1 | 1.360 (0.838‒2.207) | 1.892 (1.197‒2.990) | 0.021 |
| Model 1 | 1 | 1.682 (1.022‒2.767) | 2.491 (1.551‒4.002) | 0.001 |
| Model 2 | 1 | 1.439 (0.866‒2.390) | 2.175 (1.343‒3.522) | 0.006 |
| Model 3 | 1 | 1.099 (0.571‒2.116) | 1.377 (0.587‒3.229) | 0.716 |

Results are described as Odds Ratio and 95% Confidence Interval.

Model 1 was adjusted for age.

Model 2: Model 1 + smoking status, alcohol intake and regular exercise

Model 3: Model 2 + C-reactive protein, BMI, eGFR (baseline), DM, HTN, total cholesterol.

**Supplementary Table 3** Odds ratio of incident CKD according to leptin, adiponectin, muscle and bodyfat in women.

|  | **Q1** | **Q2** | **Q3** | **p-value** |
| --- | --- | --- | --- | --- |
| **Leptin (ng/mL)** | 36 (26.1%) | 37 (26.8%) | 65 (47.1%) | 0.002 |
| Crude OR | 1 | 1.014 (0.630‒1.630) | 1.904 (1.244‒2.916) | 0.002 |
| Model 1 | 1 | 1.091 (0.668‒1.781) | 2.068 (1.327‒3.224) | 0.001 |
| Model 2 | 1 | 1.078 (0.659‒1.763) | 2.089 (1.338‒3.263) | 0.001 |
| Model 3 | 1 | 0.875 (0.498‒1.537) | 1.197 (0.644‒2.227) | 0.518 |
| **Adiponectin** | 71 (36.6%) | 60 (30.9%) | 63 (32.5%) | 0.505 |
| Crude OR | 1 | 0.813 (0.566‒1.166) | 0.865 (0.606‒1.236) | 0.506 |
| Model 1 | 1 | 0.738 (0.506‒1.077) | 0.630 (0.433‒0.916) | 0.047 |
| Model 2 | 1 | 0.718 (0.492‒1.049) | 0.618 (0.424‒0.899) | 0.035 |
| Model 3 | 1 | 0.716 (0.467‒1.097) | 0.817 (0.529‒1.260) | 0.303 |
| **Muscle (kg)** | 86 (39.1%) | 63 (28.6%) | 71 (32.3%) | 0.126 |
| Crude OR | 1 | 0.715 (0.508‒1.006) | 0.785 (0.546‒1.093) | 0.127 |
| Model 1 | 1 | 1.048 (0.732‒1.498) | 1.549 (1.080‒2.222) | 0.038 |
| Model 2 | 1 | 1.071 (0.748‒1.533) | 1.570 (1.094‒2.254) | 0.035 |
| Model 3 | 1 | 0.947 (0.622‒1.441) | 1.117 (0.681‒1.832) | 0.764 |
| **Body fat (kg)** | 62 (28.2%) | 72 (32.7%) | 86 (39.1%) | 0.147 |
| Crude OR | 1 | 1.140 (0.800‒1.626) | 1.397 (0.992‒1.968) | 0.149 |
| Model 1 | 1 | 1.148 (0.795‒1.659) | 1.349 (0.945‒1.925) | 0.251 |
| Model 2 | 1 | 1.150 (0.795‒1.663) | 1.350 (0.945‒1.928) | 0.251 |
| Model 3 | 1 | 0.793 (0.485‒1.296) | 0.900 (0.446‒1.813) | 0.570 |

Results are described as odds ratio and 95% Confidence Interval.

Model 1 was adjusted for age.

Model 2: Model 1 + smoking status, alcohol intake and regular exercise.

Model 3: Model 2 + C-reactive protein, BMI, eGFR (baseline), DM, HTN, total cholesterol.
